# Innovations, Applications, and Future Trends in Veterinary Diagnostic Technologies

**DOI:** 10.1155/tbed/6973879

**Published:** 2026-08-02

**Authors:** Jingyu Wang, Qisheng Peng

**Affiliations:** ^1^ State Key Laboratory for Diagnosis and Treatment of Severe Zoonotic Infectious Diseases, Key Laboratory for Zoonosis Research of the Ministry of Education, Institute of Zoonosis, and College of Veterinary Medicine, Jilin University, Changchun, Jilin, China, jlu.edu.cn

**Keywords:** AI, CRISPR-Cas, high-throughput sequencing, multiplex qPCR, veterinary diagnostics

## Abstract

Veterinary diagnostics is undergoing a significant transformation driven by technological advancements, extending its scope from the traditional confirmation of specific pathogens to the continuous, dynamic surveillance of animal population’s health. This paradigm shift has the potential to enable more timely disease control, precise intervention, and enhanced public health security. Traditional clinical and laboratory diagnostic methods, such as microbial culture, serological assays, and nucleic acid‐based polymerase chain reaction, form the cornerstone of the current diagnostic framework and are widely applied based on varying detection needs and practical environments. Nonetheless, the field is experiencing profound innovation. Firstly, novel detection technologies are emerging, such as digital PCR (dPCR), CRISPR‐Cas‐based molecular diagnostic tools, next‐generation sequencing (NGS), and metagenomic sequencing. These technologies have not only achieved breakthroughs in sensitivity and specificity but, more importantly, enable the unbiased discovery of novel pathogens. Secondly, the deep integration of artificial intelligence (AI) and big data is reshaping the diagnostic pipeline. By consolidating and analyzing multimodal information streams from imaging, genomics, wearable devices, and production data, AI algorithms can provide objective, quantitative decision support, facilitating a transition from post‐symptomatic diagnosis towards predictive and preventive health management. This scoping review systematically summarizes both mainstream and emerging veterinary diagnostic technologies, elaborates and discusses their advantages and limitations as well as future developmental directions, while highlighting that the combined application of multiple methods represents an optimal diagnostic strategy.

## 1. Introduction

Pathogenic microorganisms have always been a significant factor affecting public and veterinary health. During the outbreak phase of certain pathogens or the early stages of major epidemics, timely and accurate diagnosis is crucial for effective management of infected animals and for the control of public health risks. However, there is currently a lack of comprehensive and innovative review articles covering the application of AI and big data in veterinary diagnostics. Furthermore, factors such as the frequent emergence of new infectious diseases, the rapid mutation rate of pathogens, and the high proportion of co‐infections pose significant challenges to veterinary diagnosis.

For the majority of animal pathogenic microorganisms, traditional diagnostic approaches typically include clinical and laboratory diagnoses. Clinical diagnosis is often prone to misdiagnosis due to similar symptoms, and the frequent occurrence of co‐infections involving multiple microorganisms further complicates the clinical assessment. Common laboratory diagnostic techniques, such as microbial culture, hemagglutination assays, ELISA, PCR, and histopathological analysis, are applied according to different detection needs and environments. Many novel detection methods have been developed through innovations and adaptations of these traditional approaches, and the integration or combined use of different methods has enhanced the diagnostic speed and accuracy. The rapid advancement of basic medical disciplines and foundational theories and experimental technologies in molecular biology, cytology, genetics, immunology, enzymology, nuclear medicine, and omics have created more opportunities for innovation in pathogen detection. Concurrently, the advent of the big data era and the rapid development of artificial intelligence (AI) have led to the emergence of increasingly innovative diagnostic methods, bringing new prospects to veterinary medicine.

This article focuses on summarizing the applications, advantages, and disadvantages of both commonly used and emerging diagnostic technologies in the field of veterinary medicine. It aims to provide veterinary professionals with new insights for selecting appropriate diagnostic methods under varying detection requirements and for exploring novel diagnostic technologies.

## 2. Literature Search Methodology

A comprehensive literature search was conducted using PubMed, Google Scholar, Springer Link, Wiley Online Library, and Web of Science databases for articles published primarily between 2010 and 2026. Search terms included combinations of “veterinary molecular diagnostics,” “qPCR,” “digital PCR,” “isothermal amplification,” “nucleic acid hybridization,” “high‐throughput sequencing,” “Nanopore sequencing,” “CRISPR,” “immunoenzymatic techniques,” “artificial intelligence and big data analysis technologies,” etc., along with specific pathogen names (e.g., “African swine fever virus,” “*Brucella*”). The inclusion criteria focused on original research articles, authoritative reviews, guidelines from bodies like WOAH, and cutting‐edge applications of innovative diagnostic technologies in recent years. The diseases covered are primarily those listed by the World Organisation for Animal Health (WOAH) and the “Catalogue of Class I, II, and III Animal Diseases” published by the Ministry of Agriculture and Rural Affairs of China. Articles were excluded if they were not available in English or were outside the scope of veterinary infectious disease diagnostics. This strategy ensured a focused review of the most relevant and influential advances in the field.

## 3. Pathogen Nucleic Acid Testing Techniques

**Figure 1:**
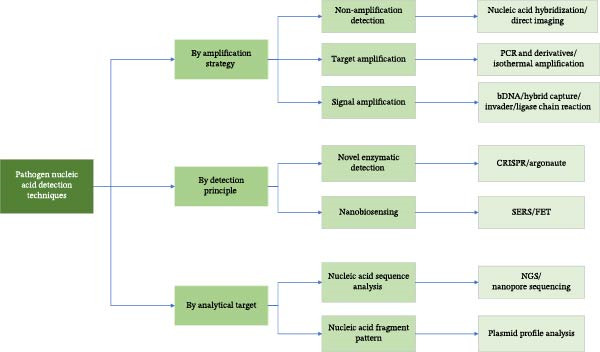
Classification of pathogen nucleic acid detection techniques.

The most notable characteristics of pathogen nucleic acid testing techniques in diagnostic techniques are high sensitivity, high specificity, and rapid detection [1, 2]. It can directly identify the genetic material of pathogens, enabling accurate diagnosis at an early stage of infection. Its core function is to break through the limitation of the traditional “culture‐before‐identification” approach, providing decisive laboratory evidence, especially when pathogens are difficult to culture, viruses mutate frequently, or emerging infectious diseases break out. In modern veterinary diagnostic systems, nucleic acid testing techniques has risen from an auxiliary tool to the “gold standard” for disease confirmation, pathogen tracing, and eradication assessment. It serves as a core technical support for building rapid response capabilities against major animal diseases (Figure 1 and Table 1).

**Table 1:** Examples of key nucleic acid testing methods.

Technology/platform	Technological innovations	Core functions and features	Typical application scenarios
Isothermal amplification	PMA‐LAMP, RPA, NASBA	No thermal cycling required; rapid reaction; simple instrumentation; ideal for on‐site use.	1. Point‐of‐care testing (POCT) 2. Pathogen screening in resource‐limited settings
qPCR	Multiplex qPCR, portable/microfluidic qPCR	Real‐time fluorescence monitoring; closed‐tube system prevents contamination; high sensitivity and quantitative.	1. Pathogen detection 2. Gene expression analysis 3. Reference “gold standard” diagnostic method
Digital PCR	Droplet digital PCR, chip‐based dPCR	Absolute quantification without a standard curve; high accuracy for rare mutations and copy number variations.	1. Detection of low‐titer pathogens 2. Liquid biopsy 3. Copy number variation analysis
CRISPR‐based diagnostics	SHERLOCK, DETECTR	High specificity; can be coupled with lateral flow for visual readout; usually requires pre‐amplification.	1. Rapid pathogen diagnosis 2. Mutation detection 3. Field‐based/point‐of‐care testing (POCT)
High‐throughput sequencing	NGS, nanopore sequencing	High throughput; enables metagenomics, whole‐genome sequencing, molecular typing, and traceability.	1. Pathogen genomics 2. Antimicrobial resistance gene analysis 3. Tracing the origin of the epidemic 4. Surveillance of emerging infectious diseases
Mass spectrometry analysis	MALDI‐TOF MS	Rapid, high‐throughput, accurate identification of microorganisms or molecular markers.	1. Microbial identification 2. Analysis of PCR products 3. Proteomics research

### 3.1. Core Amplification Technologies

#### 3.1.1. PCR Technology and Its Derivatives

In the field of veterinary diagnosis, PCR is valued for its high sensitivity and specificity. A variety of advanced derivative technologies have been developed, establishing PCR as a core diagnostic tool in veterinary medicine. The most commonly used PCR techniques today include conventional PCR, real‐time quantitative PCR (qPCR), nested PCR, and multiplex PCR. In addition to these mainstream methods, several newer PCR technologies, such as digital PCR (dPCR), high‐throughput multiplex qPCR, Nano‐qPCR, and FRET‐qPCR, have emerged as cutting‐edge tools for precision diagnosis [3–5].

##### 3.1.1.1. Conventional PCR and Nested PCR

Conventional PCR is a basic amplification technique, with results analyzed via gel electrophoresis. It is simple to perform, low‐cost, and suitable for qualitative detection of basic pathogens. It is often used in epidemiological surveys of animal diseases in laboratory settings, for example, in the epidemiological investigation of feline parvovirus (FPV) [6]. Nested PCR employs two consecutive rounds of PCR amplification cycles using two pairs of primers, greatly enhancing sensitivity and specificity. It is particularly useful for detecting samples with extremely low pathogen loads, such as in bacterial detection in laboratory animals or diagnosis of latent pathogen infections [7].

##### 3.1.1.2. Real‐Time qPCR

Real‐time qPCR incorporates fluorescent dyes or probes into the reaction system to monitor the amplification process in real time. It allows relative or absolute quantification based on cycle threshold (*C*t) values and offers high specificity and sensitivity, making it the diagnostic method with the broadest application in the veterinary field. qPCR is used for diagnosing many animal diseases, as well as for immune efficacy assessment and pathogen quantification studies [1, 8]. When using dye‐based qPCR (e.g., SYBR Green), with sufficiently optimized primer design and thorough melt curve validation, the sensitivity and quantitative accuracy are fully adequate for clinical diagnostic needs [9, 10]. Probe‐based qPCR, by incorporating a probe that adds a second layer of specificity, is more advantageous for complex samples (e.g., feces, tissue homogenates) or multiplex assays, although it comes with higher cost and design complexity [11]. Therefore, for routine diagnostics, dye‐based qPCR is fully competent and offers significant cost advantages; probe‐based methods should be prioritized for multiplex detection, trace‐level detection, or complex sample analysis [12].

A newer technique, PMA‐qPCR, combines the efficient amplification of qPCR with the ability of propidium monoazide (PMA) dye to distinguish live from dead bacteria. This makes it particularly suitable for evaluating bacterial pathogen viability in complex samples and for detecting high‐risk pathogenic microorganisms [13–15]. FRET‐qPCR employs a unique dual‐probe design: signal is generated only when both probes accurately bind to the target, achieving ultra‐high specificity. Because of its exceptional specificity, FRET‐qPCR is especially well‐suited for detecting single nucleotide polymorphisms (SNPs) and can precisely differentiate genotypes through melt curve analysis [16].

Multiplex qPCR allows the inclusion of multiple primer pairs in a single reaction system to simultaneously detect several targets, making it useful for differential diagnosis or detection of mixed infections, for example, simultaneous detection of multiple porcine epidemic viruses [17–19]. High‐throughput multiplex qPCR, through microfluidics and automation, has transformed the scale of experiments and greatly improved screening efficiency. Owing to its ultra‐high throughput, a single run can simultaneously test hundreds of samples for tens of thousands of targets, significantly saving sample and cost. For instance, in response to the complex pathogen landscape in dogs in the Asia‐Pacific region, researchers have developed a novel quadruplex qPCR capable of simultaneously detecting six major pathogens, with sensitivity superior to conventional PCR and results comparable to next‐generation sequencing (NGS), thus providing a powerful tool for epidemiological surveillance [5].

Nano‐qPCR revolutionizes the heating method using nanomaterials, achieving extreme speed and sensitivity [20]. For example, Wang et al. [21] successfully used it to detect bovine herpesvirus type 1, the causative agent of infectious bovine rhinotracheitis (IBR), achieving sensitivity surpassing that of conventional qPCR and demonstrating the ability to identify more positive cases from clinical samples.

##### 3.1.1.3. dPCR

The dPCR represents the forefront of precision detection and is a valuable early‐disease monitoring tool [2, 22, 23]. Taking brucellosis as an example, conventional qPCR struggles to distinguish attenuated vaccine strains from wild‐type strains. Recent studies using SNP‐based droplet digital PCR (ddPCR) can accurately differentiate the S2 vaccine strain from wild strains, with a sensitivity 10‐fold higher than qPCR and a detection limit as low as 10 copies/μL. This holds great potential in disease eradication and vaccine efficacy evaluation [24].

#### 3.1.2. Isothermal Amplification Technologies

Current isothermal amplification technologies can be categorized based on whether they rely on enzymatic amplification. They include loop‐mediated isothermal amplification (LAMP), recombinase polymerase amplification (RPA), nucleic acid sequence‐based amplification (NASBA), strand displacement amplification (SDA), helicase‐dependent amplification (HDA), rolling circle amplification (RCA), cross priming amplification (CPA), multiple displacement amplification (MDA), and insulated isothermal PCR (iiPCR). Owing to differences in their underlying principles and key enzymes, each technique exhibits distinct characteristics. Most share the major advantage of rapid reaction speed, making them particularly suitable for on‐site rapid detection of pathogens or food safety testing.

##### 3.1.2.1. RPA/CPA

Among these, RPA stands out for its speed and ease of use, and it is one of the most widely adopted isothermal amplification techniques. As an enzyme‐dependent isothermal method, it completely bypasses the need for high‐temperature denaturation. This allows amplification to proceed rapidly at very low temperatures (37°C–42°C), making RPA the preferred choice for developing point‐of‐care testing (POCT) tools [25]. For example, Zhai et al. [26] developed an RPA method combined with a lateral flow dipstick to detect African swine fever virus (ASFV). The entire process—from nucleic acid extraction to result interpretation—can be completed within 20 min. Results are read directly from the dipstick lines without requiring any complex instrumentation. The sensitivity and specificity of this method are comparable to those of qPCR [26].

CPA employs multiple pairs of specific primers and is mediated by Bst DNA polymerase at a constant temperature (typically 60°C–65°C). The reaction takes 45–60 min, and results can be interpreted with a dipstick in 3–5 min, making CPA suitable for basic laboratories and on‐site testing [27, 28].

##### 3.1.2.2. Hybridization Chain Reaction (HCR)

A ground‐breaking innovation at the mechanistic level is the HCR, a truly enzyme‐free isothermal amplification technique that completely eliminates the reliance on polymerases found in conventional molecular diagnostic techniques [29]. In veterinary diagnostics, HCR is still in an active phase of exploratory and applied research and has not yet become a routine diagnostic technology. Current applications focus primarily on the development of highly sensitive detection methods, with great potential, especially for detecting major animal diseases and veterinary drug residues [30, 31]. The latest PX‐HCR technology, which utilizes parallel crossover DNA structures, further improves stability in complex environments and the response speed to RNA targets. It enables real‐time observation of pathogen activity within cells, opening up possibilities for in situ imaging and live‐cell detection [32].

##### 3.1.2.3. The iiPCR

The iiPCR technology represents a successful cross‐disciplinary integration of physics and biochemistry. It achieves thermal cycling through a temperature gradient within a capillary tube on a single, constant‐temperature heating block. This elegantly combines the accuracy of PCR with the portability of isothermal equipment. Although essentially a PCR method, iiPCR simplifies the detection device, making it portable, affordable, and suitable for rapid diagnosis, primary care clinics, customs quarantine, and resource‐limited settings [33]. For instance, Song et al. developed a sensitive and user‐friendly fluorescence probe hydrolysis‐based iiPCR technique capable of detecting and differentiating two genotypes of ASFV within 40 min. Upon completion of PCR amplification, positive or negative results are automatically displayed on the device screen, providing a useful tool for on‐site detection and genotyping of ASFV [34].

##### 3.1.2.4. Loop‐Mediated Isothermal Amplification

Certain combinatorial applications of technologies have also achieved excellent results in the veterinary field. For example, PMA–LAMP combines the photochemical reaction of PMA dye with the efficient amplification of LAMP. This approach specifically excludes interference from dead bacteria and detects only viable bacteria, which is of great significance for guiding clinical antibiotic use and evaluating the effectiveness of environmental disinfection [35, 36]. RHAM is a remarkably practical and clever innovation built upon LAMP. By introducing RNase HII enzyme and specific probes, it enhances the specificity of isothermal amplification, making it particularly suitable for rapid on‐site testing scenarios that demand both high accuracy and timeliness [37, 38].

##### 3.1.2.5. Nucleic Acid Sequence‐Based Amplification

In recent years, emerging and re‐emerging RNA viruses have posed serious threats to global public health. There is an urgent need for rapid and reliable nucleic acid‐based methods to detect viral RNA. NASBA is specifically designed for RNA amplification with high efficiency. It serves as an “isothermal PCR” for RNA viruses and is especially suitable for detecting easily degradable RNA viruses [39, 40]. For example, Guoshuai et al. [41] developed a highly sensitive and easy‐to‐operate G4‐ThT‐NASBA system for detecting viral RNA without the need for labeled primers or probes. This system can detect viral RNA at concentrations as low as 2 copies/μL, is free from interference by other porcine viral RNAs, and is capable of real‐time detection of classical swine fever virus (CSFV) RNA in serum samples collected from the field within 2 h [41].

### 3.2. Nucleic Acid NonAmplification Detection Technologies—Nucleic Acid Hybridization

Nucleic acid hybridization technology holds core significance in veterinary diagnostics because it provides a detection capability that combines both “specificity” and a “spatial perspective.”

#### 3.2.1. ISH and F ISH

In situ hybridization (ISH) and fluorescence in situ hybridization (FISH) are representative DNA hybridization techniques. Using labeled probes that specifically bind to target nucleic acid sequences, they allow direct visualization of pathogen nucleic acids within cells or tissues, thereby revealing the precise location of infection. These methods are widely used for detecting viruses, bacteria, and parasites, as well as for studying tissue distribution. They provide spatial information and morphological correlation, serving as an important complement to pathological diagnosis.

ISH can be observed using a bright‐field microscope without the need for expensive fluorescence equipment, offering high cost‐effectiveness. Moreover, the signals are durable, and stained sections can be preserved long‐term for archiving and re‐examination. For example, highly sensitive BaseScope RNA‐ISH can precisely localize foot‐and‐mouth disease virus (FMDV) RNA to elucidate the tissue distribution of persistent infection, while DNA‐ISH can track the cellular localization of ASFV DNA [42, 43]. In latent or late‐stage infections where viral antigens have been cleared but nucleic acids persist, ISH offers unique advantages over immunohistochemistry (IHC).

Compared with ISH, FISH further expands applicability through its multicolor fluorescence labeling capability. It provides higher sensitivity and resolution, enabling simultaneous detection of multiple targets. However, it requires more sophisticated equipment and entails higher costs. FISH is suitable for rapid and multiplex pathogen detection and is widely used in chromosomal abnormality analysis, cancer genetics research, and co‐localization detection of multiple pathogens [44–46].

#### 3.2.2. Microarray Chip Technology

Microarray chip technology immobilizes known pathogen‐specific gene probes (e.g., from viruses and bacteria) onto a chip. These probes hybridize with fluorescently labeled nucleic acids from a sample, and pathogen identification is achieved by reading the fluorescence signals. This technology can detect thousands of targets in a single run, require minimal sample volume, and save both sample and time. When combined with automated equipment, it enables full process standardization from hybridization to result interpretation, reducing human error, while its quantitative capacity is limited [47, 48]. Microarrays are mainly used in research and epidemiological surveys, such as high‐throughput screening of multiple pathogens, genotyping, and host–pathogen interaction studies [49, 50]. For instance, Ji et al. [51] developed a diagnostic cDNA microarray for the differential detection of multiple porcine viruses, significantly improving detection efficiency. Although traditional microarrays have limited application in routine veterinary clinical diagnostics, the concept of “high‐throughput parallel detection” is being revitalized on new technological platforms.

#### 3.2.3. Multiplex Ligation‐Dependent Probe Amplification

Multiplex ligation‐dependent probe amplification (MLPA) is a multiplex detection technique for targeted nucleic acid quantification. It ingeniously combines the core technologies of “nucleic acid hybridization” and “PCR amplification” into a high‐throughput detection method. The core principle uses unique pairs of half‐probes: each probe pair can be ligated to form a complete, amplifiable template only when both hybridize adjacently to the target sequence without any gap. Subsequently, PCR amplification is performed using universal primers, and the amount of amplification product is proportional to the original target copy number. The entire procedure can be carried out on conventional PCR instruments and capillary electrophoresis systems, making it relatively simple to perform. The main advantage of MLPA is its ability to detect copy number variations efficiently and specifically. Traditional MLPA kits can simultaneously detect ~40–50 target sites in a single reaction. This technology has already been applied to the detection of animal diseases. For example, Zhou et al. [52] successfully established an MLPA method capable of simultaneously detecting and distinguishing seven porcine respiratory pathogens. This capacity for multiplex detection and differential diagnosis of mixed infections provides a reliable foundation for future big‐data sources in veterinary diagnostics.

### 3.3. Nucleic Acid Sequencing Technologies

High‐throughput sequencing (HTS) is a powerful genomics tool. HTS enables unbiased sequencing of all nucleic acid sequences present in a sample, making it particularly suitable for discovering unknown pathogens and for metagenomic analysis [53, 54]. In veterinary diagnostics, when faced with unexplained animal disease outbreaks or “syndromic” illnesses, this technology can directly sequence all nucleic acids in a clinical sample. It can not only identify novel or unexpected pathogens within a short time but also allow precise phylogenetic tracing of the pathogen [55–59]. Despite its great potential, HTS still faces multiple barriers to clinical adoption. High cost, the complexity of data analysis, and the strong dependence on specialized expertise are the main obstacles currently limiting its widespread application [57, 60].

#### 3.3.1. NGS

Current mainstream NGS technology platforms include Illumina’s SBS technology, MGI’s DNBSEQ, and Thermo Fisher’s semiconductor sequencing. Among these, Illumina SBS offers extremely high throughput, short read lengths, and high accuracy (>99.9%). Although Illumina library preparation is generally more time‐consuming and costly, leveraging its first‐mover advantage and very high throughput, the Illumina platform has long been the preferred choice for scientific research on animal diseases and for deep sequencing of complex samples. It is also the most widely used NGS platform globally [61, 62]. NGS can also be used for antimicrobial resistance gene analysis. Through metagenomic sequencing, the resistance gene profile of microbial communities can be directly analyzed from a sample, providing guidance for rational clinical use of antibiotics [56, 63]. NGS is also applicable to host–pathogen interaction studies: using transcriptome sequencing, changes in host gene expression following infection can be analyzed, revealing pathogenesis and host immune responses. These examples illustrate the cutting‐edge applications of NGS in veterinary diagnostics [64].

#### 3.3.2. Single‐Molecule Sequencing

Single‐molecule sequencing technologies (third‐generation sequencing), particularly PacBio SMRT and Oxford Nanopore, are leveraging their unique advantages, ultra‐long read lengths, real‐time sequencing, and no requirement for PCR amplification, to open up new application scenarios for animal disease detection. They demonstrate great potential in resolving complex genomic regions, discovering novel pathogens, and conducting real‐time field surveillance [65–68].

PacBio SMRT sequencing was developed earlier. In the field of animal diseases, its relatively high instrument and running costs, along with the requirement for bioinformatics expertise, limit its immediate application in resource‐limited settings. At present, it is used more as an advanced research tool to solve complex diagnostic problems, build reference genome databases, and understand the deep molecular mechanisms of diseases [65].

The portability and real‐time capability of Nanopore sequencing are its core competitive advantages, enabling even more cutting‐edge application scenarios in animal disease detection [69]. This technology is driving a shift in diagnostic capacity from central laboratories to farms, border crossings, and field sites. For example, with portable Nanopore sequencers such as MinION deployed on farms, at border inspection posts, or in wildlife surveillance stations, it becomes possible to monitor genetic variations of highly variable viruses such as Zika in real time, providing strong data support for vaccine selection and epidemic early warning [70, 71]. In resource‐limited regions, researchers have used the MinION device to reveal for the first time the diverse pathogen spectrum in local dog populations, including pathogens with zoonotic potential [71]. Although Nanopore sequencing is limited by complex library preparation workflows requiring specialized enzymes, kits, and instruments, making the process susceptible to technical variability, more accessible kits (e.g., rapid barcoding kits) are now available that reduce dependence on expensive enzymes. Thanks to its real‐time capability, portability, and information richness, Nanopore sequencing combined with real‐time analysis systems has become a powerful and disruptive tool in veterinary diagnostics [72]. It enables veterinarians and epidemiologists to shift from the traditional “hypothesis‐driven” diagnostic model to an unbiased “comprehensive exploration and precise targeting” model, representing an important future direction for the field.

### 3.4. Multifunctional Technology Platforms

#### 3.4.1. CRISPR‐Cas

CRISPR‐Cas technology represents a breakthrough in direct pathogen detection. It can detect pathogen nucleic acids at concentrations as low as a single copy per microliter, offering extremely high sensitivity. At the same time, the CRISPR RNA (CrRNA) can precisely discriminate single‐base differences, providing high specificity [73]. Combined with rapid detection speed and readout via portable fluorometers or common lateral flow dipsticks, it is highly suitable for point‐of‐care use on farms, in veterinary clinics, and other grassroots settings [74–76].

The CRISPR‐Cas technology platform mainly comprises DNA endonuclease‐targeted CRISPR trans reporter (DETECTR) and specific high‐sensitivity enzymatic reporter unlocking (SHERLOCK). DETECTR recognizes DNA, activates collateral cleavage, and cuts DNA reporter molecules. This platform offers high sensitivity and can be combined with isothermal amplification techniques such as RPA or LAMP, eliminating the need for complex precision instrumentation [77, 78]. For instance, Lin et al. [79] have developed a detection method based on RPA‐CRISPR/Cas12a, which can be used for on‐site rapid screening and epidemic monitoring of ASFV in pigs. Chen et al. [80] established a LAMP‐CRISPR/Cas12b method that provides a sensitive and specific approach for rapid detection of canine parvovirus (CPV); the results showed 100% consistency with real‐time qPCR, and the detection time was relatively short. Yao et al. [81] developed a self‐catalytic circular DNA‐driven plasmonic CRISPR/Cas12a platform. By designing specialized circular DNA, they achieved recycling of Cas12a enzyme and self‐catalytic signal amplification, increasing the sensitivity for non‐nucleic‐acid targets by ~52‐fold and reducing the reaction time to 15 min [81]. A novel effector, Gs12‐18, a homolog of Cas12a, has a broader PAM recognition range and temperature adaptability. Wang et al. [82] created a LAMP‐CRISPR/Gs12‐18 detection platform that enables visual detection of the novel duck reovirus (NDRV) S3 gene with high specificity and sensitivity.

SHERLOCK recognizes RNA, activates collateral cleavage, and cuts RNA reporter molecules. For avian influenza virus (AIV), CRISPR/Cas13a‐based detection methods targeting subtypes such as H5 and H7 have been developed, allowing rapid identification of highly pathogenic AIV [83, 84]. A novel combination of Cas12a and Cas13a leverages the distinct specificities of different Cas enzymes for DNA/RNA to enable simultaneous detection of two viruses in a single tube. For example, Guo et al. [85] developed an RT‐LAMP‐CRISPR‐Cas12a/13a‐LFD dual‐detection platform that can simultaneously detect swine influenza virus (SIV) and porcine reproductive and respiratory syndrome virus (PRRSV) (both RNA), truly achieving “one‐tube, dual‐target” detection for differential diagnosis of mixed infections with high efficiency.

At present, CRISPR‐Cas diagnostic technologies still have several limitations [86–88]. Firstly, most methods rely on nucleic acid extraction and purification from samples; this process is cumbersome, and complex components in the sample (e.g., haem, mucus) can inhibit amplification and CRISPR cleavage activity. Secondly, current techniques are predominantly qualitative or semi‐quantitative; their quantitative accuracy and reproducibility at low target concentrations are still inferior to those of qPCR [89]. Thirdly, crRNA design and reaction conditions (e.g., magnesium ion concentration, temperature, buffer system) require fine optimization for each target; otherwise, false positives or reduced sensitivity may occur. Finally, reporter probes bearing chemical modifications such as fluorophores‐quenchers or biotin are costly, and some Cas enzymes have limited ability to discriminate single‐base mismatches, making it difficult to use them directly for complex mutation typing. These issues need to be addressed to further expand the application of CRISPR‐Cas technology.

To overcome the problems of high probe synthesis cost and poor stability, scientists have developed several methods that do not rely on the aforementioned traditional probes. One example is the probe‐free CRISPR technology based on electrochemical sensors. It achieves detection by converting the CRISPR recognition event into changes in current or impedance at the electrode surface [90]. Zhou et al. [91] developed a CRISPR‐Cas13a‐based electrochemical sensor for porcine epidemic diarrhea virus (PEDV) that requires no nucleic acid amplification at all and has an extremely low detection limit, truly enabling direct “sample‐to‐result” detection. Colorimetric methods based on nanomaterial‐induced color changes have already been well established for infectious disease diagnostics in human medicine. This strategy, combining the specificity of CRISPR with the high catalytic activity of nanozymes, holds promise for application to rapid on‐site detection of major animal diseases in the veterinary field [92]. In addition, nanomaterials such as gold nanoparticles, quantum dots (QDs), DNA nanostructures, and upconversion nanoparticles have been strategically used to improve intracellular delivery of CRISPR components, facilitate signal readout, and enable multimodal sensing in complex cellular environments [93].

Given the high sensitivity of both CRISPR and microfluidic technologies, extensive research and commercial exploration have applied such platforms to the diagnosis of pathogenic microorganisms [94–96]. For example, Zhang et al. [97] integrated a CRISPR‐Cas12a system onto a microfluidic chip for specific detection of influenza A virus subtype H1N1. The method is highly sensitive and produces results within 20 min, greatly shortening the diagnostic time [97]. Xie et al. [96] developed a biodiagnostic platform named MiND‐DMF, which integrates three cutting‐edge technologies—digital microfluidics, isothermal nucleic acid amplification, and CRISPR detection—on a single chip. It enables rapid, multiplex pathogen detection under isothermal conditions, with results read out by a portable fluorescence reader, achieving full automation and miniaturization from sample to result. Overall, MiND‐DMF offers many advantages: its small size and ease of operation allow it to be used directly on farms, in veterinary clinics, or at border inspection posts, enabling “sample in, result out” and greatly reducing on‐site diagnosis time. Moreover, some DMF systems can process multiple samples in parallel, minimizing manual operation and human error, making them suitable for large‐scale disease surveillance on farms. DMF provides a novel solution for rapid on‐site testing of complex samples and holds extremely broad application prospects in veterinary diagnostics [95, 98, 99].

#### 3.4.2. Argonaute

The application of Argonaute (Ago) technology in veterinary diagnostics centers on its high specificity (single‐base resolution) and high sensitivity (signal amplification). It can be widely used for pathogen detection (especially viruses) and SNP genotyping, often in combination with isothermal amplification techniques such as RPA or LAMP to achieve rapid detection [100–103]. In recent years, following the surge of interest in CRISPR/Cas‐based molecular diagnostics, Argonaute has attracted increasing attention as a “next‐generation” or “complementary” tool for nucleic acid detection. Particularly after 2020, the number of papers on using Argonaute to detect ASFV, among other pathogens, has grown significantly. Given the large genome size and genotypic diversity of ASFV, leveraging the high specificity of Ago to avoid false negatives caused by mutations has become a research hotspot [104]. Many studies have confirmed that Argonaute‐based methods offer comparable specificity and sensitivity compared to traditional qPCR [101, 104]. However, many highly efficient Ago proteins (e.g., TtAgo) are thermophilic and require relatively high temperatures (e.g., 65°C–75°C) for optimal trans‐cleavage activity. While this enhances specificity, it also increases equipment demands. Compared to CRISPR/Cas‐based technologies, which already have commercial companies and kits available, mature commercial diagnostic products based on Argonaute technology are still very rare on the market, and researchers are actively working on their development.

#### 3.4.3. Phylogenetic Analysis

Molecular phylogenetics is a powerful approach for disease diagnosis and for understanding evolutionary relationships among organisms at the molecular level [105]. Phylogenetic methods can calibrate molecular clocks using the sampling times of molecular sequence data, thereby enabling estimation of the evolutionary rates of rapidly evolving pathogens [106]. With the development of phylogenetic methods to study pathogen evolution, Lefranc et al. [107] developed a bioinformatics tool called Phylowave. This scalable method summarizes changes in population composition within phylogenetic trees, allowing automatic detection of lineages based on shared fitness and evolutionary relationships [107]. It can quantify the ability of pathogen variants to adapt to their environment and automatically identify new variants with enhanced transmissibility or immune escape potential. The development of this tool is itself rooted in an interdisciplinary perspective; its core concept is real‐time monitoring of pathogen adaptive evolution to support prevention and control decisions. It is fully applicable to the surveillance of important animal pathogens. Although it has not yet been directly applied in specific veterinary diagnosis cases, it is highly likely to become a representative application example in veterinary epidemiology and cross‐border animal disease control in the future.

## 4. Immunological Techniques

**Figure 2:**
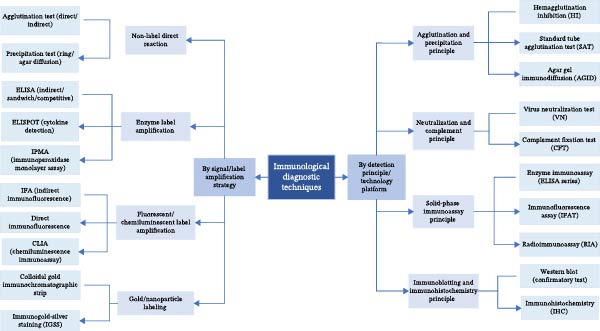
Classification of immunological diagnostic techniques.

Immunological techniques represent a core diagnostic tool in veterinary medicine. Their classification can be delineated across multiple dimensions, including the stage of immune reaction, signal labeling modality, and technical application format (Figure 2 and Table 2). Major categories encompass immunoenzymatic techniques, immunofluorescence techniques, chemiluminescence immunoassays (CLIAs), immunochromatographic techniques, immunoprecipitation/agglutination techniques, immunosensor techniques, and novel antibody/labeling technologies. As research progresses, an increasing number of innovative immunological methods continue to emerge [108, 109].

**Table 2:** Representative of cutting‐edge serological testing methods.

Technology/Platform	Technological innovations	Core functions and features	Typical application scenarios
Enzyme‐linked immunosorbent assay (ELISA)	High‐sensitivity peptide‐based ELISA, digital ELISA, nanopore‐based ELISA	High‐throughput, automatable, suitable for batch testing. Quantitative/semi‐quantitative detection of specific antibodies/antigens with high sensitivity and specificity.	1. Serological diagnosis of infectious diseases 2. Quantification of hormones and tumor markers 3. Widely used as a reference or confirmatory method
Chemiluminescence immunoassay (CLIA)	Magnetic microparticle‐based CLIA, acridinium ester/alkaline phosphatase systems	Ultra‐high sensitivity, broad dynamic range, and precise quantitative capability. Supports full automation for rapid and high‐throughput testing.	1. Trace‐level analyte quantification 2. Early diagnosis and therapeutic monitoring of infections 3. High‐sensitivity detection of tumor markers, hormones, and biomarkers
Immunofluorescence assay (IFA)	Indirect IFA, time‐resolved fluorescence immunoassay (TRFIA)	Uses fluorescent labels for precise spatial localization of targets, enabling qualitative to semi‐quantitative detection with high specificity.	1. Confirmation and subtyping of pathogens 2. Autoimmune disease diagnostics 3. Cellular/tissue localization of specific antigens
Immunochromatographic assay (ICA)	Lanthanide/europium nanoparticle‐based ICA, quantum dot‐based ICA	Rapid, user‐friendly, qualitative or semi‐quantitative point‐of‐care screening. Offers good sensitivity and stability; results can be interpreted visually or with portable readers.	1. Rapid clinical screening 2. Preliminary diagnosis in resource‐limited or veterinary settings 3. Herd health monitoring and field epidemiological surveys

### 4.1. Immunoenzymatic Techniques

This platform is currently the most extensively utilized in veterinary diagnostic laboratories, with its core advantage residing in signal amplification achieved through enzymatic catalysis. Principal examples include Enzyme‐linked immunosorbent assay (ELISA) and Enzyme‐linked immunospot assay (ELISPOT).

#### 4.1.1. ELISA

ELISA stands as the most widely applied technology within immunoenzymatic methods. It is characterized by technical maturity, relatively low cost, high throughput, and ease of standardization and automation. Monoclonal antibody‐based ELISA constitutes the absolute workhorse of veterinary laboratory diagnostics [110, 111]. Primarily operating through formats such as sandwich ELISA and competitive ELISA, it is well‐suited for large‐scale sample screening and remains the method of choice for diagnosing numerous diseases and monitoring antibody responses [111–113].

Since its inception, the ELISA technology has undergone continuous innovation and transformation. Although conventional ELISA facilitates rapid testing of multiple samples, limitations in detection sensitivity and result accuracy have persistently constrained its further development. High‐sensitivity ELISA represents an advancement over traditional ELISA, exemplified by high‐peptide ELISA, chemiluminescent ELISA, and nano‐signal amplification ELISA. These formats typically employ highly sensitive chromogenic or chemiluminescent substrates, or signal amplification systems, thereby achieving superior specificity and sensitivity in detecting neutralizing antibodies [114–116]. As the foundation of current mainstream automated platforms, they serve as the primary source of stable, reliable, and comparable standardized data for routine large‐scale analysis.

Beyond high‐sensitivity peptide‐based ELISA, ultra‐high‐sensitivity formats include electrochemical ELISA, digital ELISA (dELISA), and Nanopore‐based signal readout ELISA [117]. These approaches overcome the inherent limitations of conventional ELISA and have progressively demonstrated enhanced performance in practical applications. For instance, electrochemical ELISA converts conventional colorimetric or fluorescent signals into measurable electrochemical signals for quantification. Its core advantages include ultra‐high sensitivity, potential for portability, and low‐cost feasibility [118]. Currently, these technologies are primarily in the stages of laboratory proof‐of‐concept and prototype development. When combined with nanomaterial‐modified electrodes, further improvements in sensitivity and stability are anticipated.

Paper‐based ELISA, owing to its low cost, minimal equipment requirements, and operational simplicity, is particularly suitable for POCT. With technological advances, colorimetric or fluorescent readouts can be captured using smartphone cameras and quantitatively analyzed via dedicated applications, positioning it as a vital diagnostic tool for resource‐limited settings [119]. Such techniques function as “terminal data collectors” within the broader data ecosystem rather than core data generators. They are responsible for rapidly acquiring critical information at farms or clinics and uploading these structured data to the cloud in real time, thereby enhancing the coverage breadth and temporal resolution of the big data network [120, 121].

dELISA achieves single‐molecule sensitivity and represents a current frontier in the field. The Simoa platform is the most commercially successful implementation of dELISA, boasting sensitivity up to 1000‐fold greater than conventional ELISA and finding widespread application in detecting ultra‐low‐abundance biomarkers in areas such as infectious disease surveillance [122, 123]. This technology provides longitudinal data of exceptionally high sensitivity, making it ideally suited for long‐term, precise monitoring of a select panel of critical, low‐abundance markers, thereby contributing high‐quality time‐series data points to big data analytics, albeit with inherently limited throughput [124]. A recently published study described the integration of magnetic bead arrays, microfluidics, tyramide signal amplification (TSA), and AI–based image recognition to develop an “open‐surface digital ELISA (OS‐dELISA)” platform. Applied to the direct detection of ASFV p30 antigen, this assay achieved a detection limit as low as 21 fg/mL within a mere 17 min, offering a powerful tool for ultra‐early, on‐site diagnosis [125].

Nanopore readout ELISA, which employs nanopores to detect enzymatic reaction products in lieu of conventional colorimetry, has demonstrated detection limits reaching the attomolar (aM) range, representing a sensitivity improvement of three to four orders of magnitude over chemiluminescence, hereby showcasing a paradigm shift in signal acquisition strategies [126]. To date, this technology has not yet been applied to veterinary disease diagnostics.

Conversely, liquid‐phase ELISA modifies the traditional paradigm of reactions confined to solid surfaces. It enables ultra‐high‐throughput multiplexed detection with exceptional specificity and sensitivity and has already gained widespread application in veterinary diagnostics [127–129]. Collectively, these diverse innovations in ELISA provide robust technical support for obtaining timely and accurate diagnostic results both in the laboratory and at the point of care.

#### 4.1.2. ELISPOT

According to the latest research advances, the application of ELISPOT technology in laboratory animal disease diagnosis is steadily progressing from the stage of scientific exploration toward practical implementation; however, its scope of application remains largely concentrated in specific niches rather than serving as a universal, routine diagnostic tool [130, 131]. Unlike antibody detection techniques such as ELISA, the core value of ELISPOT lies in the assessment of cellular immune responses. It enables the enumeration, at the single‐cell level, of cells secreting specific cytokines (e.g., IFN‐γ), achieving a detection sensitivity as high as one in a million cells [132]. ELISPOT can also be employed for the early diagnosis of important zoonotic diseases. For instance, a newly developed IFN‐γ ELISPOT assay for *Brucella* infection by Wang et al. demonstrated a sensitivity of 96.9% and a specificity of 90.6%, with a detection sensitivity ~30‐fold higher than that of the corresponding ELISA method [130]. More importantly, this study confirmed that ELISPOT is capable of detecting infection earlier than traditional serum agglutination tests and offers a prolonged diagnostic window period. Currently, the application of ELISPOT in laboratory animal disease diagnosis is positioned as a “supplementary, high‐level tool for precise scenarios” rather than a routine screening modality [132].

### 4.2. Immunofluorescence Techniques

Immunofluorescence techniques constitute a core methodology that integrates immunological specificity (antigen–antibody reaction) with fluorescent labeling to detect, trace, and localize target proteins, pathogens, and other biomolecules. The results are visually intuitive, rendering these techniques suitable for qualitative or semi‐quantitative analysis. In direct immunofluorescence, the fluorophore is conjugated directly to the primary antibody, which binds to the target antigen. This approach offers rapid processing and high specificity, albeit with relatively lower sensitivity. In contrast, indirect immunofluorescence employs an unlabeled primary antibody to bind the antigen, followed by detection with a fluorophore‐conjugated secondary antibody. This method provides higher sensitivity and is more commonly utilized [133, 134]. In addition to conventional fluorophores such as FITC, immunofluorescence labels now encompass novel materials including QDs and lanthanide chelates.

#### 4.2.1. Immunofluorescence Histochemistry/Cytochemistry and Fluorescence in Situ Hybridization

Immunohistochemical and immunocytochemical techniques based on organic fluorescent dyes such as FITC and Alexa Fluor dyes offer notable advantages in terms of high specificity and sensitivity. Crucially, they provide essential spatial information regarding the distribution of target molecules within cells or tissues, making them invaluable for localizing pathogens and investigating pathogenic mechanisms in precision veterinary diagnostics [133]. Their limitations include relatively complex procedures, the requirement for expensive fluorescence microscopy, and susceptibility to interference from nonspecific staining, rendering them less suitable for applications with high background or demanding precise quantification. For single‐target detection, FITC presents a clear cost advantage; for prolonged observation or photographic documentation, optimized dyes such as Alexa Fluor 488 yield superior performance [135].

FISH is a nucleic acid hybridization technique based on complementary base pairing. It utilizes labeled nucleic acid probes that bind specifically to DNA or RNA target sequences within intact tissue sections or cell samples, thereby enabling direct visualization of the precise spatial distribution of target nucleic acids within their morphological context. In veterinary diagnostics, FISH is particularly adept at elucidating the specific site and status of infection. With rationally designed probes, FISH enables simultaneous detection of multiple targets and is widely applied in pathogen co‐localization studies [44, 46, 136]. Multiplex fluorescence imaging, as an extension of the aforementioned techniques, facilitates simultaneous multi‐target imaging using multiple organic dyes. This approach has been extensively employed in veterinary diagnostics and research, including disease screening, pathological investigations, and immunological analysis [137, 138]. However, its procedural complexity and time‐consuming nature preclude its utility for on‐site rapid screening.

#### 4.2.2. Flow Cytometry

Flow cytometry represents a high‐throughput, multiparametric quantitative analytical technique widely used in veterinary clinical settings. It employs fluorophore‐conjugated antibodies to recognize specific antigens on the cell surface or intracellularly, enabling rapid analysis of large cell populations and precise identification and enumeration of distinct cellular subsets. While flow cytometry plays a central role primarily in basic research and in the diagnosis and classification of certain noninfectious diseases, recent years have witnessed its increasing application in the early diagnosis of animal infectious diseases, demonstrating high sensitivity and specificity [139, 140].

#### 4.2.3. Fluorescence Immunochromatographic Test Strips

Immunofluorescence techniques employing QDs as labels, which are characterized by high fluorescence intensity and exceptional photostability, can significantly enhance on‐site detection sensitivity and enable simultaneous detection of multiple targets on a single test strip. For instance, a QD–based lateral flow strip for detecting H5 subtype AIV achieved a sensitivity of 0.0625 HAU with 100% specificity [141].

#### 4.2.4. Time‐Resolved Fluorescence Immunoassay

Immunofluorescence techniques employing time‐resolved fluorescent microspheres (TRFIA) as labels offer excellent signal stability and are unaffected by ambient light interference, enabling “zero‐background” detection. These attributes are primarily exploited in high‐sensitivity fluorescence immunochromatographic strips and TRFIA, particularly suited for applications demanding exceptional sensitivity and accurate quantification. For example, the detection limit for *Brucella* antibodies can reach 0.048 IU/mL using this approach, surpassing traditional methods by several orders of magnitude [142]. Furthermore, a TRFIA‐based immunochromatographic strip for detecting Pseudorabies virus (PRV) gB antibodies exhibited an overall concordance rate exceeding 95% with ELISA [143]. Li et al. [144] developed a dual‐label TRFIA for the detection of canine coronavirus (CCV) and canine parvovirus type 2 (CPV‐2) antigens, enabling differentiation of the two viruses in a single test.

In summary, FITC remains the “workhorse” for laboratory IHC/cytochemistry, indispensable for histopathological research and mechanistic investigations of pathogenesis. QDs exhibit clear advantages in fluorescence immunochromatographic test strips, particularly suited for multiplex detection and rapid on‐site screening. When maximal sensitivity (e.g., early infection) or precise quantification is paramount, TRFIA or flow cytometry represents the method of choice.

### 4.3. CLIA

CLIA primarily encompasses direct CLIA, enzyme‐catalyzed chemiluminescence immunoassay (CLEIA), and electrochemiluminescence immunoassay (ECLIA). Among these, CLEIA involves labeling an antigen or antibody with an enzyme (e.g., HRP, ALP). Following the antigen–antibody reaction, a specific chemiluminescent substrate for the enzyme (e.g., luminol, AMPPD) is added, and the enzyme catalyzes a chemical reaction that generates a luminescent signal. This format is widely employed in veterinary diagnostics [145–147]. A currently advanced technique in this domain is a magnetic particle‐based chemiluminescence technology, which uses magnetic beads as solid‐phase carriers. It offers a large surface area, high reaction efficiency, and easy washing steps, requires no external light source for excitation, and exhibits low background noise [148, 149]. Owing to its advantages of high sensitivity, broad linear range, rapid detection speed, and full automation capability, it is particularly well‐suited for large‐scale screening, precise surveillance, and eradication assessment of major animal diseases and has become a mainstream mid‐to‐high‐end immunoassay platform in clinical diagnostics and life science research [149]. For instance, Wang et al. [150] developed an automated CLIA for the quantitative and qualitative detection of ASFV p72 antibodies, which significantly reduces assay time and labor‐intensive steps while improving detection sensitivity and linear range.

ECLIA represents a specialized form of chemiluminescence wherein the luminescent reaction (e.g., involving ruthenium bipyridine labels) is triggered by applying a voltage to an electrode surface. Because the reaction is precisely controlled at the electrode interface, background interference is exceptionally low, the linear range is extremely broad, and result reproducibility is excellent. ECLIA is renowned for its high precision and minimal background noise; however, due to the elevated cost of instrumentation and reagents and its relatively closed technology ecosystem, its application is primarily confined to high‐end diagnostic platforms and human diagnostic fields [151, 152]. Recently, Canadian scientists have successfully applied ECLIA for the detection of antibodies against highly pathogenic AIV H5N1. Notably, this assay can detect antibodies not only in serum but also in dried blood spot samples, and it effectively discriminates cross‐reactivity between H5 and other influenza subtypes [153].

Conventional chemiluminescence relies on sophisticated instrumentation, which restricts its utility in field settings. A study published in 2025 described a smartphone camera‐based chemiluminescence lateral flow assay for the detection of ASFV antibodies. This method integrates the convenience of a test strip with the high sensitivity of chemiluminescence, enabling result readout via mobile phone photography and achieving a sensitivity enhancement of at least two orders of magnitude over conventional colloidal gold test strips [154]. Furthermore, the utilization of nanomaterials to amplify chemiluminescent signals, thereby further augmenting detection sensitivity, remains a prominent area of current research.

### 4.4. Immunochromatographic Techniques

Immunochromatographic techniques constitute a highly practical rapid detection method and play a pivotal role in the diagnosis of animal diseases. The core principle of immunochromatography relies on the specific binding between antigen and antibody. These assays are characterized by operational simplicity, requiring no sophisticated instrumentation and minimal operator training. Results can typically be interpreted visually within minutes to 20 min, and the low per‐test cost facilitates widespread adoption. Consequently, they are exceptionally well‐suited for POCT in grassroots settings such as livestock farms and veterinary clinics, serving as indispensable tools for preliminary screening, on‐site rapid diagnosis, and epidemiological investigation of animal diseases [155]. Based on the nature of the label employed, the primary technical variants include colloidal gold‐based technology, QD–based technology, and techniques utilizing labels such as fluorescent microspheres and latex microspheres, each possessing distinct characteristics tailored to specific diagnostic requirements [155, 156].

The first‐generation immunochromatographic technology is predominantly represented by immunocolloidal gold techniques. Owing to its simplicity, rapid turnaround, intuitive visual readout, and low cost, it has become an essential tool for on‐site rapid testing, enabling veterinarians and livestock producers to establish an initial diagnosis of animal diseases promptly [157–160]. However, conventional immunocolloidal gold assays are constrained by limited throughput and are suboptimal for the rapid screening of large sample volumes. They also suffer from insufficient sensitivity, posing a risk of false‐negative results during early infection stages or when pathogen loads are low [161]. Moreover, these assays are primarily qualitative or semi‐quantitative in nature, precluding accurate determination of pathogen load or antibody titers. To address these limitations, researchers have continually refined methodologies to enhance sensitivity and specificity [162, 163]. For instance, Zhang et al. [164] developed a dual‐target colloidal gold test strip capable of specifically identifying antibodies against both the p30 and CD2v proteins of ASFV, thereby enabling the rapid differentiation of variant strains.

The second‐generation immunochromatographic technology is typified by fluorescence immunochromatography. This approach utilizes fluorescent substances, such as organic fluorescent dyes or lanthanide chelates, as labels. These materials do not inherently emit light but fluoresce upon excitation by a specific wavelength light source, with the resultant fluorescence intensity measured by a dedicated instrument. The principal advantages of fluorescence immunochromatography lie in its exceptionally low background interference and high sensitivity, leading to its widespread application in veterinary diagnostics [155, 165, 166]. Among these, lanthanide‐based fluorescence immunochromatography represents a cutting‐edge direction in POCT, integrating the high sensitivity of lanthanide fluorescent labels with the rapidity and portability of lateral flow strip technology. For example, Zhang et al. [167] established a rapid, sensitive, specific, and reproducible europium nanoparticle‐based immunochromatographic strip (EuNPs‐ICTS), which serves as a promising tool for the on‐site diagnosis of *Toxoplasma gondii* infections in dogs and cats.

The third‐generation immunochromatographic technology, exemplified by QDs, upconverting nanoparticles, and fluorescent nanospheres, aims to address the demands for greater speed, enhanced accuracy, and higher multiplexing capacity. These platforms pursue ultra‐high sensitivity, simultaneous multi‐analyte detection, and improved resistance to interference, representing the current focal point of research and development [168–170]. In a recent study, Li et al. [156] pioneered the use of quantum dot microspheres (QDMs) simultaneously labeled with recombinant ASFV proteins P30 and P54 as detection probes. The integrated system, comprising a QDM immunochromatographic strip and a handheld fluorescence immunoassay analyzer, enabled ultrasensitive and quantitative detection of ASFV antibodies in serum [156]. Another investigation reported the first development of a QD–based duplex immunochromatographic strip, featuring two test lines (T1 and T2) on a single strip for the simultaneous detection of antibodies against ASFV p54 and CD2v. This advancement not only confirms infection but also permits differentiation between highly virulent wild‐type infection and infection with a low‐virulence CD2v‐deleted strain [171]. Furthermore, nano‐enhanced immunochromatographic technology replaces conventional colloidal gold with nanoparticles exhibiting superior luminescent properties, enabling quantitative detection through fluorescence intensity measurements. The continuous introduction of novel nanomaterial labels and optimization of fabrication processes promise further upgrades and expansion in sensitivity and quantitative performance [172–174]. With the advent of QD and broader nanotechnology applications, immunochromatographic platforms are steadily transcending their conventional limitations.

## 5. Signal Readout and Amplification Technologies

### 5.1. Nanobiosensing Technologies

Nanobiosensing technologies, predominantly those based on localized surface plasmon resonance (LSPR), surface‐enhanced Raman scattering (SERS), and field‐effect transistors (FETs), are providing novel solutions for the prevention and control of zoonotic diseases and for companion animal diagnostics. LSPR‐based technology exhibits relatively high technical maturity and distinct cost advantages and has already been applied to the detection of viral antigens and veterinary drug residues [175]. SERS technology excels in pathogen and virus detection owing to its ultra‐high sensitivity and molecular fingerprinting capability. For example, Song et al. developed a SERS‐based immunochromatographic test strip incorporating gold‐coated magnetic nanoparticles, exploiting the enrichment effect of magnetic nanoparticles and the high sensitivity of SERS to achieve rapid on‐site differential diagnosis of co‐infections with porcine epidemic diarrhea virus (PEDV) and porcine rotavirus (PoRV) [176]. FET technology, as a rapid, portable, and electrically transduced platform, demonstrates considerable potential for on‐site differential diagnosis of viral infections. For example, PEDV S protein‐specific monoclonal antibodies can be immobilized onto aurum nanoparticles (AuNPs) modified on a floating‐gate carbon nanotube FET chip. When the virus binds to the antibody, the resulting charge changes can be amplified in real time by the FET and converted into electrical signal [177]. Interface engineering approaches (e.g., DNA nanotechnology) advanced in the human medical field are expected to overcome current bottlenecks limiting FET application in complex biological samples [178].

### 5.2. Direct Imaging and Nanoparticle Counting

In contrast to conventional methodologies such as ELISA or PCR, ultra‐sensitive detection techniques that operate at the single‐molecule or single‐particle level through physical or optical means of “direct imaging and nanoparticle counting”—exemplified by NanoPick‐array, digital p‐FLISA, and mSATORI—enable ultra‐early, high‐precision, and absolute quantitative outcomes in animal disease diagnostics. Presently, these technologies remain predominantly at the stage of laboratory prototype development; nevertheless, they exhibit remarkable innovation in specific directions. Their capacity for multiplex detection and performance in real‐world complex sample matrices constitute critical challenges that must be addressed in future advancements [179, 180].

## 6. Omics Technologies

In the diagnosis of animal pathogenic microorganisms, omics technologies have demonstrated formidable capabilities. Not only can they accurately identify pathogens, but they also provide deep insights into the interaction mechanisms between hosts and pathogens, offering novel perspectives and tools for disease surveillance, diagnosis, prevention, and control [181].

### 6.1. Genomics and Metagenomics

Genomic technologies, particularly metatranscriptomics and metagenomic sequencing, depend on next‐generation or third‐generation sequencing technologies (e.g., Illumina or nanopore). These approaches enable the culture‐independent, one‐time detection of all microorganisms (e.g., viruses and bacteria) present in a sample, essentially establishing an “identification card” for pathogens. Therefore, these technologies are primarily used for identifying pathogen species, conducting taxonomic classification, and further analyzing genetic evolution and tracing transmission sources through gene sequence analysis. A nationwide study covering pig farms across 25 provinces in China utilized this technology to map the “pathogen panorama” of porcine respiratory diseases. It precisely identified the Porcine Reproductive and PRRSV as the “primary culprit” and uncovered potential threats from emerging viruses such as Getah virus [182].

### 6.2. Transcriptomics

Transcriptomics dynamically reflects changes in gene activity during infection, revealing the pathogenic strategies of pathogens and the host’s immune response mechanisms. Beyond simply indicating which genes are switched on or off, transcriptomics captures the magnitude, timing, and co‐regulation patterns of gene expression changes during infection. This finer resolution allows researchers to distinguish between primary pathogenic events and secondary host responses. Van et al. systematically demonstrated that transcriptomics facilitates the early diagnosis of *Mycobacterium avium* ssp. *paratuberculosis* (MAP) infection by identifying transcriptional signatures at different stages of infection to develop novel diagnostic markers [183].

### 6.3. Proteomics

Proteins are the direct executors of life activities. Proteomics, based on technologies such as mass spectrometry or antibody microarrays, enables the discovery of key proteins associated with infection by comparing protein expression differences between healthy and diseased animal samples (e.g., tissues, body fluids, or cells) in the context of veterinary infectious disease diagnosis, thereby facilitating the identification of reliable diagnostic biomarkers. Zhou et al. successfully screened three proteins (HSPA8, B2M, and HRG) from extracellular vesicles in the plasma of cows infected with *Mycobacterium bovis*. These proteins hold promise as potential markers for developing simpler on‐site detection methods for bovine tuberculosis [184].

MALDI‐ToF mass spectrometry based on bacterial isolation and culture is a powerful technique for the rapid identification of microorganisms, and it also has a wide range of applications in the detection of antimicrobial resistance [185]. Its core principle involves “weighing” microbial proteins to generate unique spectral “fingerprints,” which are then compared against databases for identification [186]. The advantages of MALDI‐ToF MS include rapid detection, high throughput, low operational cost, and high accuracy. However, this technique currently remains dependent on prior isolation of pure cultures and cannot be directly applied to raw clinical samples. Its efficacy is also reliant on the comprehensiveness of reference databases; consequently, its ability to identify rare or emerging pathogens that are absent from these is limited, and it may lack sufficient discriminatory power for closely related subspecies or serotypes. Despite its current dependence on cultivation, researchers are actively working to overcome this limitation. This technology has substantially enhanced the efficiency of microbial identification in veterinary diagnostic laboratories [187, 188].

### 6.4. Metabolomics

Metabolomics detects small molecule metabolites, the end products of life activities. Since infection rapidly induces perturbations in metabolic networks, metabolomics holds unique advantages for early diagnosis. A study on *Escherichia coli* septicemia in broiler chickens found that serum adenine levels decreased significantly within just 8 h post‐infection. This makes it a highly promising early warning indicator, potentially advancing the diagnostic window substantially [189].

### 6.5. Multi‐Omics Integration and Future Trends

Current research frontiers are no longer confined to the application of single omics technologies but are increasingly inclined toward integrated analysis of genomics, transcriptomics, proteomics, and metabolomics. This “multi‐omics” approach constructs a more comprehensive “panorama” of infection from different levels, thereby systematically elucidating pathogenic mechanisms. For instance, in dissecting the infection process of bovine coronavirus (BCoV), scientists integrated transcriptomic and proteomic data and found that 48 h post‐infection, most differentially expressed genes and proteins were significantly enriched in biological processes such as metabolic pathways and immune responses to acquire energy for viral replication, while also revealed a novel mechanism by which the virus downregulates the key complement system component C3 to achieve immune evasion [190].

## 7. AI and Big Data Analysis Technologies

AI and big data analysis technologies are penetrating every aspect of animal healthcare, bringing profound changes to traditional veterinary diagnosis. They not only enhance the efficiency and accuracy of diagnosis but also demonstrate great potential in early disease detection, precise judgment, and scientific prevention and control (Table 3).

**Table 3:** Representatives of artificial intelligence and big data analysis technologies.

Technology/platform	Core functions and features	Typical application scenarios (veterinary diagnostics focus)
Deep learning medical image analysis	Automatically analyzes medical images using deep neural networks to identify, segment, and classify lesion areas.	1. Diagnosis of thoracic diseases in livestock 2. Cardiac screening in pets 3. Automated blood smear analysis and parasite detection
AI intelligent diagnosis system/multimodal large model platform	Integrates image recognition, NLP, and knowledge graphs for multi‐source data analysis, enabling rapid screening and decision support.	1. Preliminary disease screening based on clinical signs 2. Remote veterinary consultation 3. Development of farm‐specific biosecurity and testing protocols
Integrated smart detector (with PCR and microarray chip)	Integrates extraction, amplification, and detection in a portable device for automated “sample‐in, result‐out” testing.	1. On‐farm high‐throughput pathogen detection 2. Animal genotyping for breeding and disease resistance 3. Rapid screening for import/export quarantine
Macro‐omics data analysis platform	Integrates multi‐omics data and uses AI/bioinformatics tools to identify biomarkers and construct regulatory networks.	1. Molecular tracing and evolutionary analysis of animal diseases 2. Ppathogen identification and function prediction 3. Screening of molecular markers
Intelligent sensing and real‐time monitoring system	Uses biosensors, IoT, and wireless transmission for real‐time monitoring of physiological/environmental parameters, with early‐warning algorithms.	1. Real‐time herd health monitoring on farms 2. Early warning for disease outbreaks 3. Environmental monitoring and biosafety alerting in animal facilities

### 7.1. Image Recognition and Analysis

Image recognition and analysis can utilize deep learning models to automatically analyze medical images, assisting in the identification of lesions and prognosis judgment. The integration of deep learning‐based tools into diagnostic workflows is increasingly prevalent due to their efficiency and reproducibility in various settings [191]. Glahn et al. [192] developed deep learning algorithms to automatically analyze the nuclear morphology of canine lung cancer cells and predict the prognosis of the disease. The method can simultaneously handle multiple morphological parameters such as nuclear area and nuclear intensity and analyze a large number of cell nuclei within seconds. This high‐throughput, multi‐parameter analysis capability is beyond the reach of manual operation [192].

The rapid advancement of digital slide visualization has propelled pathology into a new era—the era of digital pathology (DP) [193]. It works by scanning entire tissue sections into high‐resolution digital images, uploading them to cloud platforms, and applying AI algorithms to automatically analyze these digital slides, identifying specific pathological patterns (such as intranuclear inclusion bodies and distinct inflammatory cell types) and even directly detecting pathogens [194]. This enables remote diagnosis, quantitative analysis, and high‐throughput screening, thereby reducing subjective human error and improving diagnostic efficiency and consistency [195].

### 7.2. Multimodal Data Fusion

Multimodal data fusion can integrate various sources of information such as text, images, and sensor data to construct a comprehensive diagnostic model. Wen et al. proposed a multimodal PGID diagnosis method based on AI and large language models (LLM). ChatGPT and image augmentation techniques were used to expand the dataset, and the multi‐scale TextCNN model was employed to capture multigranularity semantic features from text. By integrating text cases and intestinal image features, they conducted intelligent diagnosis of porcine gastrointestinal infectious diseases. The cross‐modal RF classification achieved an accuracy rate of 87.58% [196], thus demonstrating high performance. The multimodal approach provides an efficient and accurate intelligent solution for diagnosing PGID, outperforming single‐modal methods.

### 7.3. Macroscopic Omics Data Analysis

Utilizing AI to analyze omics data such as metagenomics and proteomics for pathogen identification and function prediction. Li et al. [197] improved the YOLOv8n model (real‐time object detection algorithm) and developed the YOLO‐Cocci model (the YOLO model for coccidia), which can automatically detect chicken coccidia oocysts in vaccine environments, with mean average precision at intersection over union threshold 0.5 (mAP@0.5) reaching 89.6%, an increase of 6.5% compared to the benchmark model. The model enhances detection accuracy while reducing both model parameters and computational cost. They further developed client software that supports automated detection and provides visualization of the results.

Despite advances in pathogen detection using deep learning, current algorithms have limitations in processing long‐read genomic sequences. To address the challenge of accurately identifying pathogenic bacteria contamination in complex environmental microbial communities, Jiang et al. [198] developed the DCiPatho deep learning model through the deep cross‐fusion of cross, residual and deep neural networks, which can be used to accurately identify distinct pathogenic bacteria infecting animals based on the entire genome sequence, with an identification accuracy of up to 95.14%. Thus, DCiPatho serves as an effective tool for the genomic‐scale identification of pathogens.

### 7.4. Intelligent Sensing and Real‐Time Monitoring

The integration of Internet of Things (IoT) devices and AI algorithms enables round‐the‐clock health monitoring and early warning for both individual animals and herds. Verde et al. developed an AI‐driven thermal imaging system, integrated with robotic milking, that automatically and accurately segments udder regions, extracts the maximum skin temperature, and eliminates environmental interference to ensure the reliability of the temperature data, achieving early and noninvasive detection of subclinical mastitis in Italian Mediterranean buffalos [199]. In pig farming, intelligent track inspection robots and devices such as the “Cough Monitor” can be utilized to collect environmental data and physiological indicators from pigs [200, 201]. Through continuous monitoring, analysis, and AI model evaluation of this data, early warnings for respiratory diseases and other health issues can be achieved [202].

## 8. Discussion

### 8.1. Selection of Diagnostic Methods

The global expansion of livestock farming scale and density, coupled with the gradual degradation of biodiversity, has led to an unprecedented diversification and complexity of animal diseases [53]. In the prevention and control of animal epidemics, the lack of effective diagnostic methods or the inconsistency and impracticality of diagnostic guidelines are some of the key reasons why many diseases remain endemic with persistently high incidence rates. Given the current frequent outbreaks of various viral, fungal, bacterial, and parasitic diseases in the veterinary field, accurately predicting epidemic trends, achieving early detection of outbreaks, and precisely identifying pathogens are crucial for formulating targeted control strategies and reducing the risk of disease transmission. Consequently, the development of reliable, highly sensitive diagnostic and detection methods is imperative.

Routine diagnostic methods for animal diseases primarily encompass pathogen identification, serological testing, and nucleic acid‐based diagnostic techniques. Building upon these core approaches, a steady stream of optimized and novel technologies continues to emerge. Concurrently, innovations in foundational platform technologies, including probe‐based techniques, microarray technology, nanotechnology, and microfluidics, have been ingeniously integrated into nucleic acid detection and immunoassay platforms [52, 96, 203]. This convergence has propelled the rapid advancement of veterinary diagnostic technologies, while simultaneously posing considerable challenges for veterinary professionals in selecting the most appropriate diagnostic methods.

According to World Health Organization guidelines, the ideal method for detecting foreign pathogens should be rapid, specific, sensitive, instrument‐free, and cost‐effective [77]. However, given the vast diversity of animal diseases and the varied settings in which diagnosis is performed, ranging from laboratories and farms to points of entry and border inspection stations, the specific diagnostic technology chosen ultimately depends on the particular diagnostic requirements. Where ultrasensitive and precise quantification is paramount, nucleic acid detection methods such as qPCR are the preferred choice [16]. When rapid on‐site results are required, portable lateral flow strips and certain isothermal amplification techniques represent ideal options [26]. For unknown or complex pathogens, NGS offers the most comprehensive solution [56]. When striving to balance convenience with diagnostic accuracy, the combined use of isothermal amplification and CRISPR‐based detection represents a key direction for future development [77]. For large‐scale laboratory‐based sample testing where stability and cost control are prioritized, ELISA and related commercial kits remain a reliable choice [117]. When the objective is to obtain highly sensitive, more accurate results or to acquire diagnostic data rapidly at the point of need, CLIA and its various innovative formats, such as magnetic particle‐based chemiluminescence and lanthanide‐based fluorescence immunochromatography, warrant greater attention and application [150].

In selecting diagnostic methods, differentiating infected from vaccinated animals (DIVA) is a core technical challenge in animal disease control. The available options can be broadly grouped into genetic, serological, and cell‐mediated immune markers. Genetic approaches exploit genomic sequence differences between wild‐type strains and marker vaccines, such as qRT‐PCR for CSF and multiplex PCR targeting *gD* and *gE* genes for bovine infectious rhinotracheitis (IBR), offering speed and high sensitivity, yet they depend heavily on the development and widespread adoption of the corresponding marker vaccines [204, 205]. Serological approaches detect antibody responses to antigens deleted in marker vaccines; for example, the Erns antibody AlphaLISA for CSFV achieves 100% specificity and high sensitivity, works with both serum and oral fluid, and thus facilitates large‐scale field surveillance [127]. Cell‐mediated immune approaches discriminate infection by measuring cytokine release in response to virulence‐specific antigens, as with the RCE‐IGRA for bovine tuberculosis, enabling reliable DIVA distinction even in the absence of genetic markers [206]. Each approach has its strengths and suitable contexts; selecting the optimal DIVA strategy based on disease characteristics, vaccine type, and testing conditions is therefore essential for precise diagnosis and science‐based animal disease control.

### 8.2. Selection of Diagnostic Criteria

When discussing the selection of a certain diagnostic method, the determination of diagnostic criteria is indispensable. Its importance extends well beyond technical considerations, directly affecting the reliability of test results, the success of disease control, and ultimately the survival of the livestock industry [207, 208]. Regardless of the setting or the diagnostic method used, correct criteria are the cornerstone of an effective animal disease prevention and control system.

Accurate diagnosis is the prerequisite for science‐based control and the basis for interrupting transmission chains. Errors in criterion selection can lead to false positives and unwarranted panic culling or false negatives that allow outbreaks to spread. At the same time, large‐scale farming imposes greater demands on diagnostics [208]. A well‐designed set of criteria supports early detection and on‐site rapid testing, buying valuable time for containment [209]. The common industry challenge of inconsistent results from the same sample tested by different methods or kits can be effectively resolved through unified standards, ensuring accuracy and reliability [208].

For most major infectious diseases, pathogen isolation and identification remains the undisputed confirmatory gold standard, despite being time‐consuming and technically demanding [207]. Classical immunological techniques, such as agglutination tests and virus neutralization tests (VNT), continue to serve as gold standards for many significant diseases [210, 211]. In routine practice, however, a larger proportion of diagnostic standards rely on PCR or ELISA. In particular, real‐time qPCR, with its high sensitivity, specificity, and speed, has become the core laboratory technology and the preferred gold standard for an increasing number of major diseases [212].

As science and technology advance and diagnostic techniques evolve, the establishment of diagnostic criteria must not only satisfy extremely high specificity and sensitivity but also address emerging scientific questions. For example, when diagnosing emerging pathogens for which no ready‐made “gold standard” exists, statistical confirmation through latent class models is required. By combining multiple assays and complex statistical models, a relatively reliable “gold standard” can be retrospectively validated [213, 214].

### 8.3. AI and Big Data‐Driven Revolution in Veterinary Diagnostics

Current veterinary diagnosis has evolved beyond the mere identification of specific pathogenic microorganisms or confirmation during disease outbreaks; it has advanced to the level of dynamic surveillance. This shift is crucial for the timely detection of outbreaks, controlling the spread of infections, and enabling prompt treatment. The breakthrough brought by the development of big data and AI is key for meeting the demands for more accurate, efficient testing, and analysis.

Traditionally, veterinary diagnosis often relied on post‐symptomatic examination after animals displayed obvious signs. Today, the deployment of intelligent sensors and AI algorithms on farms enables real‐time health monitoring [215, 216]. Second, the paradigm has shifted from “experience‐dependent” to “data‐driven.” Traditional diagnosis heavily relied on the personal experience and knowledge of veterinarians, while AI models can integrate multidimensional data to provide objective, quantitative decision support [192]. Third, the approach has moved from “general protocols” to “precision diagnosis and treatment.” Big data analytics allows for more personalized treatment plans [217]. Furthermore, by analyzing metagenomic data, AI can accurately identify pathogenic bacteria within complex microbial communities and even discover novel pathogens, providing targets for precise prevention and control [218].

Despite the promising prospects, the comprehensive application of AI and big data in veterinary medicine faces multiple challenges. The primary issue lies in data quality and sharing: the scale of high‐quality, accurately annotated veterinary data is limited, and data silos among institutions are prevalent, constraining the training of robust models. Additionally, many deep learning models function as “black boxes,” with opaque decision‐making processes that undermine veterinarians’ trust in and clinical adoption of AI conclusions [217]. Moreover, the deployment and maintenance costs of intelligent sensing devices and AI analysis platforms are high, and there is a need for interdisciplinary professionals who possess both veterinary and technical expertise [219]. This poses a practical barrier for many farms and primary care clinics. Future development in the field will emphasize the deep integration of multimodal data, such as combining genomics data with clinical imaging to provide a more comprehensive diagnostic view. Simultaneously, privacy‐preserving computational technologies like federated learning offer the potential to break down data silos, enabling multiple institutions to collaboratively train more powerful and generalizable models while rigorously protecting data privacy.

### 8.4. Combined Application of Multiple Technologies

With the growing complexity of animal diseases, the rapid, sensitive, and specific detection of live pathogens remains a critical priority for quality control and risk assessment. Conventional methods often involve complex procedures, expensive reagents, specialized laboratory equipment, and are time‐consuming, rendering them unsuitable for field testing and resource‐limited settings. Increasingly, detection modalities integrate different sensing mechanisms to synergistically capture multidimensional information, achieving functional integration and data fusion. Such multimodal sensing technologies play a vital role in veterinary diagnostics by providing complementary information, enhancing accuracy, and enabling continuous monitoring. Meanwhile, the concept of dynamic monitoring is increasingly shaping animal disease diagnosis. For example, Nanopore sequencing coupled with real‐time analysis systems, combined with host transcriptomics, viral metagenomics, and microbiome analysis, enables rapid species‐level identification and real‐time diagnosis, thereby facilitating a comprehensive understanding of disease pathogenesis and outbreak source tracking [61]. With its speed, portability, and information richness, this integrated analytical platform is emerging as a powerful disruptive tool in veterinary diagnostics.

For major animal diseases and zoonoses, it is essential to enhance awareness and education among health professionals, improve the recognition of disease‐related clinical manifestations, and refine diagnostic strategies through the integration of multiple technologies. Leveraging big‐data platforms and AI‐driven algorithms will strengthen surveillance, while the standardization of diagnostic technical references for specific diseases is vital to ensure accurate and timely diagnosis and an effective public health response.

## Funding

This study was funded by the National Natural Science Foundation of China (Grant 32470195).

## Disclosure

All authors have read and agreed to the published version of the manuscript.

## Conflicts of Interest

The authors declare no conflicts of interest.

## Data Availability

No new data were created or analyzed in this study.
